# Supplementary material for: Are carbon emissions trading and green financial instruments synergistic? -Comprehensive quantitative research based on content analysis
Source: PLoS One. 2024 Mar 7;19(3):e0298601. doi: 10.1371/journal.pone.0298601 (PMC10919601; doi:10.1371/journal.pone.0298601)
Supplement: S1 Data — (ZIP) [file pone.0298601.s001.zip › Data/地级市Z_F工作报告.zip!/╕ú╜¿/╒─╓▌╩╨/└┤╫╘╣1⁄2╓┌║┼í╛┬φ┐╦╩2╛▌═°í┐_2011.docx]

各位代表：

　　现在，我代表漳州市人民政府向大会作政府工作报告，请各位代表连同《漳州市国民经济和社会发展第十二个五年规划纲要（草案）》一并审议，并请市政协各位委员和其他列席人员提出意见。

　　一、2010年和“十一五”经济社会发展回顾

　　过去的一年，在省委、省政府和市委的正确领导下，在市人大的法律、工作监督和市政协的民主监督下，市政府认真贯彻党的十七大、十七届三中、四中、五中全会和胡锦涛总书记来闽来漳考察重要讲话精神，深入贯彻落实科学发展观，认真实施国务院《关于支持福建省加快建设海峡西岸经济区的若干意见》，按照省委八届九次、十次全会和市委九届十次、十一次全会的部署，突出“海西建设、漳州先行”发展主线，全力推动科学发展、跨越发展，全面完成了市十四届人大五次会议确定的各项目标任务。全市地区生产总值1400.7亿元，增长14.9%；财政总收入139.4亿元，增长22.5%，其中地方级财政收入88.6亿元，增长24.8%；全社会固定资产投资837.1亿元，增长44.5%；农业总产值450.1亿元，增长4.3%；工业增加值540.5亿元，增长21.7%，工业总产值2156亿元，增长30.5%，其中规模工业产值1945.5亿元，增长33.4%；外贸出口50.7亿美元，增长49.7%；实际利用外资7.1亿美元，增长27.4%；社会消费品零售总额444亿元，增长18.1%；居民消费价格总水平上涨3.4%；城镇居民人均可支配收入18482元，增长11.2%；农民人均纯收入7861元，增长11.4%；城镇登记失业率2.6%；人口自然增长率6.1‰。单位生产总值能耗预计下降2.6%，化学需氧量、二氧化硫排放量分别控制在2.69万吨和2.5万吨以内，顺利完成节能减排目标。

　　一年来的主要工作和成效是：

　　（一）打好“五大战役”，跨越发展基础进一步夯实

　　投资大幅增长。“五大战役”成效显著，固定资产投资增幅位居全省首位。重点项目建设战役完成投资222亿元，完成计划120.7%；新增长区域建设战役完成投资143.3亿元，完成计划112.9%；城市建设战役完成投资25亿元，完成计划143.6%；小城镇改革发展战役完成投资33.8亿元，完成计划110.1%；民生工程战役完成投资20.8亿元，完成计划127.8%。古雷石化启动项目完成投资超百亿元；福欣特钢进入全面建设阶段；旗滨玻璃第三条生产线建成投产，第四条生产线开工建设。全市交通基础设施完成投资79.4亿元，完成计划107%。古雷炼化一体化、漳州军民合用机场等一批重特大项目前期工作有序推进。全市546个项目获中央、省预算内资金补助8.7亿元。

　　引资成效显著。大干150天期间，全市新签约亿元以上项目132个，总投资1923亿元，其中10亿元以上项目29个，总投资1787.6亿元。成功引进正新橡胶、联盛纸业、圣莉雅环保壁纸、康师傅饮品等一批重大项目。全年累计新签约亿元以上项目228个，总投资超过2440亿元，其中10亿元以上项目43个，总投资超过2130亿元。新批注册合同外资10.2亿美元，增长30.4%。企业增资76家，增资额2.5亿美元。第十四届中国投资贸易洽谈会签约项目当年合同外资履约率62.2%，创历史新高。对接央企和大型国企成效显著，签约10个项目，总投资579亿元。

　　作风明显改善。大力开展“经济增长看幅度、项目建设看进度、战役组织看力度”和“比拼干劲、比拼举措、比拼效率、比拼形象、比拼贡献”活动，积极倡导“一线工作法”、“五加二”、“白加黑”，坚持每月召开一次现场点评会，营造比学赶超、竞相发展的浓厚氛围。着力破解项目建设难题，制定出台项目审批、招投标以及勘察、设计、施工图审查等提速措施，推进审批提速、服务优化。行政审批项目减少108项，项目审批基本时限从110多个工作日压缩到40个工作日以内。创造出“旗滨速度”，成为全省宣传推广的典型。

　　（二）转变发展方式，产业转型升级进一步加快

　　现代农业加快发展。粮食总产量70.8万吨，完成造林24.3万亩。农产品出口21亿美元，增长55%，其中水产品、花卉出口分别增长95%、52%。农业产业化进程加快，142家市级以上龙头企业实现产值246亿元，增长28%。年产值10亿元以上的农业龙头企业由3家增加到6家，世界级食品检验实验室诺安食品安全中心落户平和。建成32个蔬菜生产示范基地，新增绿色食品10个、有机食品5个、无公害农（水）产品认证40个。加快农民专业合作社建设，2家列入农业部示范项目，86家与大型超市建立产销对接。农业“五新”推广步伐加快，测土配方施肥实现全覆盖。引导农村土地流转30万亩，实施土地整理4.3万亩，补充耕地2.2万亩，建成农业标准化示范基地20万亩，新扩建标准渔港11个，改造标准化渔塘1.1万亩。加快推进“十大水利工程”和“百个水利项目”建设，修复病险水库42座，完成海堤除险加固38.3公里。推进农村饮水安全工程建设，解决18个乡镇、20.5万人饮水安全问题。动植物疫情疫病防控得到加强，农产品质量安全监管机制不断完善。扶贫开发继续加强，库区移民后扶工作扎实推进。

　　工业发展上新台阶。实施工业“千百工程”项目1110个，完成工业投资335亿元，增长71.5%，有496个项目建成投产。新增亿元企业107家，总数达到372家，亿元企业产值1397.2亿元，增长31.6%。“4+4”重点产业产值1305.2亿元，增长32.6%，其中石化增长37.6%，钢铁增长16.4%，装备制造增长41.9%，食品工业增长45.9%。出台《关于进一步支持和推动企业上市的意见》，有20家企业列入省级重点上市后备企业。漳龙实业有限公司成功发行6亿元中期票据。“蓝火计划”对接技术转让项目120个，11个项目进入高新技术创业中心。节能照明和智能家电被确定为国家级高新技术产业化基地，新认定高新技术企业34家，高新技术产业产值460亿元，增长26%。专利授权量1520件，增长92.6%。设立市政府质量奖，新增中国驰名商标4件、福建著名商标44件、福建名牌35件。

　　第三产业发展提速。第三产业增加值523.4亿元，增长11.2%。漳州商贸集团顺利组建，苏宁电器、大润发商场开业运营，浦口省级粮食储备库建成投入使用，海峡两岸（国际）农产品物流城、海峡花卉（漳州）集散中心、明发商业广场、红星美凯龙、人民广场欢乐城、吉马汽车城、闽南汽车交易城、华安佳具群家具交易城建设提速，万达商业广场动工建设，角美奥特莱斯现代产业服务园正式签约，6家高星级酒店动工建设。“万村千乡”市场工程加快建设，建成7个农村集贸市场、6个农资配送中心、666家“农家店”。金融业加快发展，新增1家分行、6家支行，漳州农村商业银行正式创立，年末各项贷款余额836.2亿元，增长27.5%。旅游业加快发展，旅游总收入突破100亿元。商品房销售面积和销售额分别增长67.4%、97.8%。

　　（三）突出对台合作，对外开放水平进一步提升

　　载体平台有效拓展。出台《关于推进开发区新一轮创业的若干意见》，明确“三年倍增、六年跨越”的发展目标，促进开发区更好更快发展。漳州招商局开发区升格为国家级经济技术开发区，华安工业集中区升格为省级开发区，漳州台商投资区通过国家部委联审，古雷台湾石化产业园区获批设立，南太武滨海新区上升为全省发展战略，九龙生态经济区规划建设加快推进，台湾农民创业园扩区步伐加快。台湾行业公会（商会）入驻海西联络处扩展到24家，在全省率先成立闽台农业标准化研究推广中心，东海岸保税仓储物流中心赴台设立全省首家办事处。成功举办第二届农博会•第十二届花博会，有1217家国内外采购商前来洽谈订货，达成订货协议金额超过25亿元，并签约合同项目63个，总投资6.1亿美元。东山东海岸公共保税仓一期投入运营，国家级科技兴贸光电基地和农副产品及加工品、家用电器及钟表两个省级出口基地建设全面启动。

经贸合作纵深推进。胡锦涛总书记莅漳看望台商效应不断显现，先后组织34批、430人次上岛开展推介招商，特别是去年5月组织19个分团319人随省团赴台开展经贸文化交流，洽谈对接98个项目，其中签约27个项目，总投资6.7亿美元，采购金额2.6亿美元，推动漳台石化、光电等一批产业深度对接，促进正新橡胶、捷波电子、汉晶光电等一批项目落地建设。对台出口4.6亿美元，增长79.2%。

交流交往持续深化。成功举办第二届海峡论坛•开漳圣王及部属后裔回漳谒祖等一系列对台交流活动。漳台货运直航和对台快件业务不断扩大，台胞来漳旅游超过7万人次。漳台乡镇对口交流形成三年规划，22对乡镇实现对接。基层党际交流深入开展，文化、教育、卫生、宗教、民间信仰、政法、工会、妇女等各个领域的交流交往更加密切。

　　（四）注重统筹协调，城乡建设力度进一步加大

　　中心城市品位提升。行政中心东移正式获国务院批准。编制完成《漳州市中心城市拓展概念规划》、《漳州城市中心区城市设计》、《漳州城市建设整体风貌特色控制规划》、《漳州市九龙江南岸地区发展战略规划》、《漳州九龙生态经济区发展概念规划》等一系列规划，启动《漳州市城市总体规划（2010—2030）》修编，完成23个乡镇、235个行政中心村规划编制。中心市区23个城建重点项目完成投资19.6亿元。碧湖生态园建设加快，完成投资8.9亿元，征地1063.4亩。九龙江大桥、火车南站站前广场及市区主干道路加快推进。江滨公园一期基本建成，新建改建8座垃圾中转站、30座公厕，新增公交105辆。市区内河整治有力推进，截污管道铺设进展顺利。巩固提升创卫成果，城市人居环境进一步改善。

　　县域经济活力显现。八县一市规模工业增加值、财政总收入、固定资产投资等主要经济指标增长强劲，呈现竞相发展好势头。龙海县域经济基本竞争力位居全国第69位，并连续15年保持全省县域经济实力“十强”，长泰、东山、南靖、漳浦入围全省经济发展“十佳”，龙海、长泰入围全省首届科学发展“十优”。大城关建设步伐加快，县城功能配套进一步完善。角美、岩溪2个省级和11个市级小城镇综合改革建设试点有序推进，总体规划和重要专项规划基本完成，城乡建设用地增减挂钩等政策有效实施，一批启动项目开工建设。

　　新农村建设深入推进。全面完成15个“绿色乡镇”、40个“乡村公园”、123个省级“绿色村庄工程”、121个市级村容整洁示范村建设。开展16个乡镇、251个村家园清洁行动重点治理，农村垃圾治理率乡镇达到83%、村庄达到82.9%。新建农村公路“上衔下延”433公里，改造危病桥74座。完成户用沼气池1.8万户，带动农村配套改厕5596户。

　　（五）加强环境保护，生态名市建设进一步深化

　　节能减排任务全面完成。严格落实节能减排责任制，抓好节能减排重点工程建设，南靖列入首批国家级绿色能源示范县，9家企业实施清洁生产。提前实现每个县（市）至少建成一座城镇污水处理厂目标，市区、县城污水处理率分别达88%、79.9%。深入实施工业废水深度治理与工业废气脱硫工程，推进重点污染源在线监控设备安装，主要污染物排放量控制在省政府下达的指标之内。

　　环境综合整治持续推进。实行水环境治理责任制，深入抓好“两江两溪”水环境综合整治，巩固提升九龙江流域水环境整治成果，确保群众饮水安全。工业园区及农村环境连片整治取得新成效，各县（市、区）垃圾处理场全部建成，蒲姜岭生活垃圾焚烧发电厂动工建设。开展“加强环境执法监管年”活动，严厉查处企业违法排污行为，维护群众环境权益。实施“碧海银滩”行动，重点海湾环境得到有效整治。

生态修复建设得到加强。加强城乡造林绿化工作，强化自然保护区、湿地管理。实施水源、家园、田园三个示范工程，15个乡镇、20个村分别通过省级环境优美乡镇、省级生态村验收。加强矿产、岸线、海域等资源开发保护，连续12年实现耕地占补平衡。

　　（六）着力改善民生，人民生活水平进一步提高

　　防抗台风全面胜利。面对三次强台风正面袭击，我市始终突出把人民群众生命安全放在第一位，始终确保干部与群众奋战在一起，始终做到严密部署、注重细节，始终坚持全民动员、人人防抗，各级领导深入一线、靠前指挥，广大干部在岗到位，驻漳部队官兵冲锋在前，人民群众奋力自救，各项防抗措施有效落实，夺取了零伤亡、少损失的全面胜利，得到中央和省委、省政府充分肯定，市委、市政府被省委、省政府记一等功，漳浦县荣获“全国防汛抗旱先进集体”称号。

　　社会保障不断完善。启动新型农村养老保险试点工作，城乡低保对象实现应保尽保，残疾人等弱势群体基本生活得到妥善安置。新增城镇职工养老保险1.4万人、医疗保险2.5万人。城镇居民医保补助和补偿标准提高，参保人数77.7万人。农村新型合作医疗参合率99.1%，住院补偿标准继续提高。龙海率先实现城乡居民养老保险和基本医疗保险一体化。全面完成渔船和渔工保险。新开工建设廉租住房2013套，发放廉租住房租赁补贴6811户。建设经济适用房1588套，实施公共租赁住房1162套。新增就业5.9万人，下岗失业人员再就业1.4万人，新增农村劳动力转移就业8.6万人。九龙江连家船民历史遗留问题得到有效解决。

　　社会事业全面进步。26个为民办实事项目完成投资26.1亿元，占年度计划130.4%。各级各类教育协调发展，义务教育均衡发展取得新进展，学前教育进一步加强，城镇中小学扩容工程步伐加快，全市高中阶段毛入学率85%，漳州职业技术学院通过国家示范性高职建设项目验收，漳州城市职业学院通过国家高校办学水平评估，南靖一职校升格为国家级重点职校。投入9.4亿元建设中小学校安工程项目440个。提前完成基层医疗卫生机构基本药物零差率销售覆盖面达到60%的省定任务，开工建设县级医院、乡镇卫生院等基层医疗项目52个，加快建设市第二医院、龙文医院以及市中医院病房大楼、市传染病院门诊综合楼。全市实现白内障无障碍市。新建14所乡镇敬老院、30个农村幸福园、60个居家养老服务站。闽南文化生态保护实验区建设成效明显，漳州历史街区被评为中国历史文化名街，完善改造21个省级乡镇综合文化站，新增170个农家书屋。人口计生工作成效显著，荣获全国婚育新风进万家活动示范市及全国人口和计划生育综合改革示范市称号。体育事业加快发展，成功获得第十五届省运会承办权。有线数字电视整转工作继续走在全省前列，农村电影放映工程全面落实。对口援建四川省彭州市敖平镇、三界镇，投入灾后重建资金2.6亿元，突出民生优先，落实管理优化，确保工程优质，实现三年任务两年完成目标。新一轮援藏援疆援宁工作扎实推进。第六次人口普查顺利开展。精神文明建设、人事编制、妇女儿童、老干老龄、老区建设、地方志、民族宗教、外事侨务、新闻出版、统计、物价、档案、地震、气象、残联、红十字会等各项工作都取得新成效。

　　社会保持安定稳定。综治责任制有效落实，“平安漳州”建设成效明显，建立维稳信息员、网络舆情引导员、维稳群众工作队“三支队伍”，基层综治信访维稳中心作用有效发挥，公众安全感满意率95.7%，综治考核名列全省第3名。实行领导干部接访和机关干部带案下访，全市接访来访人次下降14.8%。高度重视校园安全工作，加强校园及周边安全隐患排查。狠抓安全生产专项整治，强化安全隐患源头治理，安全生产四项控制指标全面下降。严格食品药品安全监管，食品放心工程有效推进。国防动员、优抚安置、双拥共建、民兵预备役、海防人防、应急管理以及打假、反走私等工作继续加强。

　　（七）坚持依法行政，政府自身建设进一步加强

　　自觉接受人大及其常委会监督，依法执行人大决定决议，认真办理人大代表建议和意见，积极配合人大常委会开展执法检查、人大代表视察及专项调研。重视和支持人民政协履行政治协商、民主监督、参政议政职能，认真办理政协提案，积极采纳委员调研成果。全年共办理市人大代表建议155件、市政协提案275件，办结率均为100%。广泛听取各民主党派、工商联、各人民团体、无党派人士和社会各界人士意见。深入贯彻国务院《全面推进依法行政实施纲要》、《关于加强市县政府依法行政的决定》和《关于加强法治政府建设的意见》，行政执法责任制全面推行，行政决策程序更加健全，规范行政权力运行工作全面推开。“五五”普法活动全面完成，行政复议工作得到加强，政府信息公开制度深入推进，政府门户网站逐步完善。深化机关效能建设，完善政府绩效评估，强化“重在提请、主动服务”，加强市行政服务中心和市公共资源交易中心建设，政府服务发展、服务群众的质量进一步提升。严格执行党风廉政建设责任制，认真落实《党员领导干部廉洁从政若干准则》，加强政府系统反腐倡廉建设，推进惩治和预防腐败体系建设，强化行政监察、审计监督，坚决纠正损害群众利益的不正之风，政风行风进一步好转。

　　2010年工作任务的全面完成，为“十一五”规划画上了圆满的句号，为漳州“十二五”跨越发展奠定了坚实的基础。

　　过去的五年，是我市发展实力持续提升的五年。地区生产总值、人均地区生产总值五年年均分别增长13.9%、13.3%，总量均比2005年翻一番以上。工业总产值连续突破1000亿元、2000亿元两个大关，是2005年的2.8倍。财政总收入净增88.8亿元，年均增长22.4%，其中地方级年均增长25.7%。

　　过去的五年，是我市发展动力显著增强的五年。全社会固定资产投资五年累计2424.2亿元，是“十五”的3.1倍，超过1949年至2005年的总和，2010年当年投资量超过“十五”五年累计投资总和。实际利用外资五年累计41.6亿美元（可比口径），是“十五”的1.2倍。工业投资五年累计完成1079.9亿元，是“十五”的3.5倍。先后引进建设古雷石化、旗滨玻璃、福欣特钢3个投资超百亿元特大工业项目和一批投资上10亿元工业项目，这些项目都将在“十二五”期间建成投产，形成强劲发展支撑。

　　过去的五年，是我市发展方式加快转变的五年。生态名市建设全面推进，生态环境质量保持全省前列，节能减排目标全面完成。新增中国驰名商标32个，福建省著名商标145个、福建省名牌179个。第三产业增加值年均增长14.1%。三次产业结构从2005年的22.8∶40.4∶36.8优化为18.2∶44.4∶37.4。

　　过去的五年，是我市发展条件明显改善的五年。开工建设高速铁路里程262公里、高速公路里程278公里，新增农村水泥路3365公里，现代化综合交通体系日益完善。新增港口吞吐能力766万吨，全市港口吞吐量达到4300万吨。电网建设累计投资49.1亿元，新增110千伏及以上变电容量927万千伏安。新增超临界燃煤装机60万千瓦，风力装机18.1万千瓦。累计投入180多亿元用于市政公用设施建设，自来水普及率、燃气普及率、污水处理率、垃圾处理率、人均道路面积明显提高。

　　过去的五年，是我市发展成果惠及人民的五年。累计实施106个为民办实事项目。全面实现义务教育“两免一补”。城镇居民人均可支配收入、农民人均纯收入年均分别增长11.8%和10.9%。累计新增城镇就业人员28万人、实现下岗再就业7万人、转移农村劳动力40.4万人。累计建设各类保障性住房9806套。城乡低保实现应保尽保，社会保障体系不断完善。住房、汽车、旅游、教育、保健等新兴消费持续升温，人民群众小康生活水平明显提升。

　　“十一五”发展成就来之不易。这五年，我们不但克服了严重自然灾害和国际金融危机的严峻挑战，而且积累了攻坚克难、跨越发展的宝贵经验，这是我们不断战胜困难、夺取更大胜利的重要精神财富。回顾五年来不平凡的历程，我们深刻体会到：推动漳州科学发展、跨越发展，必须始终扭住第一要务，持续强化项目带动，不断增强发展后劲；必须始终把握发展机遇，持续强化机遇意识，不断拓展发展空间；必须始终坚持先行先试，持续破解各种困难，不断释放发展潜能；必须始终突出民生优先，持续改善群众生活，不断强化发展合力；必须始终做到奋发有为，持续开展创先争优，不断提升发展实效。

　　过去五年取得的成绩，是省委、省政府和市委正确领导的结果，是全市人民团结奋斗的结果，是方方面面大力支持的结果。在此，我代表市人民政府向辛勤工作在各行各业的广大干部群众，向给予政府工作大力支持的人大代表、政协委员、各民主党派、工商联、离退休老同志和社会各界人士，向积极服务漳州发展的中央、省驻漳单位和驻漳部队、武警官兵，向为漳州发展做出突出贡献的企业家、投资者和建设者，向关心支持漳州发展的港澳台同胞、海外侨胞和国际友人，表示衷心的感谢并致以崇高的敬意！

　　在看到成绩的同时，我们也清醒地认识到发展中还存在不少困难和问题，主要有：综合实力仍然不强，产业结构不尽合理，重大项目还不够多；农业农村基础设施仍然较为薄弱，农民持续增收难度加大；财政收支矛盾较为突出，民生改善和社会建设投入有待进一步增加；中心城市辐射带动能力不强，城镇化进程有待进一步推进；社会保障体系不够健全，文化、教育、医疗卫生等公共服务还不能满足群众需求；安全生产、社会治安存在一些薄弱环节，社会管理有待加强；政府职能需要加快转变，一些工作人员服务意识不强、工作效率不高、破解难题办法不多，个别领导干部出现腐败现象。对这些问题，我们一定要高度重视，认真加以解决。

　　二、精心实施“十二五”规划，开创漳州跨越发展新局面

　　“十二五”是漳州加快转变经济发展方式、推动跨越发展的重要战略机遇期。我们要抓住一切可用机遇，调动一切积极因素，集中精力抓发展，全力以赴促跨越，奋力开创现代化建设新局面，提前五年建成全面小康社会。

　　根据党的十七届五中全会、省委八届十次全会和市委九届十一次全会精神，市政府组织编制了《漳州市国民经济和社会发展第十二个五年规划纲要（草案）》（以下简称《纲要（草案）》），提请本次大会审议。《纲要（草案）》总结了“十一五”规划的实施情况，分析了“十二五”的宏观环境和面临的挑战，提出了今后五年经济社会发展的总体要求、目标任务和重大措施。

　　《纲要（草案）》明确我市“十二五”发展的指导思想是：高举中国特色社会主义伟大旗帜，以邓小平理论和“三个代表”重要思想为指导，深入贯彻落实科学发展观，紧紧围绕“海西建设、漳州先行”，坚持以科学发展、跨越发展为主题，以加快转变经济发展方式为主线，以深入实施“依港立市、工业强市、开放活市、科教兴市”战略为重点，以项目带动、产业提升和区域突破为支撑，以保障和改善民生为立足点，着力推进农业现代化、新型工业化和城镇化进程，着力推进城乡一体化发展和区域协调发展，着力推进对台交流合作先行先试和改革开放纵深发展，努力推动经济社会在新的起点上实现跨越发展，提前五年全面建成小康社会，把漳州建成海峡西岸生态工贸港口城市。

　　《纲要（草案）》提出了经济发展、民生发展、科教发展、可持续发展四大类36项主要目标，其中约束性13项、预期性23项。明确地区生产总值年均增长14%以上，到2015年总量达 3000亿元以上；财政总收入到2015年总量达400亿元以上；综合实力进入全省先进行列。这些发展目标，体现了跨越发展、加快赶超的要求，注重发展速度、质量和效益相协调，突出调整经济结构和保障改善民生，经过全市人民共同努力是可以实现的。

《纲要（草案）》分17个章节提出了今后五年经济社会发展的工作重点和主要任务，归纳起来有以下几个方面。

　　（一）突破重大项目，充分发挥投资引领作用。全力引进建设一批重大产业支撑项目，促进产业发展层次明显提升。统筹推进综合交通、能源、市政公用、信息网络和防灾减灾等基础设施建设，加快构建适度超前、功能配套、抗灾和应急能力强、安全高效的现代化基础设施体系。集中力量建设事关发展、惠及民生、展示形象的“双十工程”。加快一批重大项目前期工作，为跨越发展提供源源不断的后续支撑。

　　（二）突破重点区域，全力打造经济新增长极。围绕加快建设厦漳泉大都市区，实施市区“中心东移、跨江南扩、面海拓展”发展战略，加快行政区划调整，做大做强中心城市，增强带动辐射能力。全力突破古雷港经济开发区、南太武滨海新区、漳州台商投资区、九龙生态经济区建设，积极推进东山生态旅游岛建设，统筹发展其他重点开发区和工业集中区，引导重大产业项目、各种优势资源向重点区域集聚发展，推动产业、要素、人口有效集聚，加快形成若干重要的新经济增长区域。

　　（三）突破重点产业，加快构建现代产业体系。着力建设海西先进制造业基地，力争到2015年规模工业产值突破7000亿元。加速壮大石油化工、特殊钢铁、装备制造、食品工业四大主导产业，到2015年四大主导产业产值均超过1000亿元。培育发展电子信息、新材料、新能源、生物与新医药四个战略性新兴产业，力争产值年均增长30%以上。积极应用高新技术和先进适用技术改造提升家具、钟表、造纸及纸制品、金属制品、建筑材料和船舶修造等传统产业。推动金融、物流、旅游等现代服务业成为主导产业，大力发展信息、商贸、服务外包和家庭服务业。加快发展海洋产业，建设海洋经济强市。

　　（四）突破三农问题，深入建设社会主义新农村。提升现代农业发展水平，加快农业规模化、产业化、集约化、现代化进程。完善现代农业体系，稳定粮食生产能力，做精做深八大农业特色产业，加快推进“一县一业”、“一县一品”，加速推广农业“五新”，大力发展种子产业，加强农业品牌创建、保护和宣传推介，打造“中国食品名城”。拓宽农民增收渠道，努力增加农民经营性、工资性、财产性和转移性收入。改善农村生产生活条件，强化以水利建设为重点的农村基础设施建设，加快农村社会事业发展，全面提高防灾减灾能力，创新农村发展体制机制。

　　（五）突破关键环节，全面增强经济发展活力。着力打造漳台合作交流创新平台，推进漳台产业深度对接、文化深度交流、民间深度交往，努力构建两岸交流合作的重点先行区。实施更加积极主动的开放战略，强化载体建设，优化外资结构，提升利用外资质量和水平。持续拓展外贸出口，推进对外贸易从规模速度型向质量效益型转变，实现外贸市场结构、产品结构多元化。加快重要领域和关键环节的改革步伐，努力构建充满活力、富有效率、更加开放、有利于科学发展的体制机制。

　　（六）突破环境保护，持续推进生态名市建设。积极构建资源节约型、环境友好型社会，大力推进生态名市、森林城市建设。强化水仙花原产地、九湖万亩荔海和天宝万亩蕉林保护，规划建设水仙花公园、荔海公园和蕉林公园。扶持发展绿色经济、低碳经济和循环经济，强力推进节能减排，降低能源消耗，减少污染排放。着力解决人民群众最关心、最突出的环境污染问题，高度重视水环境安全，强化城乡饮用水源保护，加快市区第二水源建设。

　　（七）突破社会建设，提升人民群众幸福指数。加快推进以改善民生为重点的社会建设，让发展成果更好惠及广大人民群众。坚持教育优先发展，加快普及从学前到高中的十五年教育，加快人才强市建设步伐，增强科技创新能力。实施更加积极的就业政策，完善覆盖城乡居民的社会保障体系，不断提高城乡居民收入水平。改善居民住房条件，保障居民基本住房需求。加快建设文化强市，大力发展文化事业，加快发展文化创意产业。深化医药卫生体制改革，提高人民群众健康水平。注重优生优育优教，提高人口素质。加强和创新社会管理，切实维护社会和谐稳定。

　　三、扎实做好2011年工作，实现“十二五”良好开局

　　2011年是“十二五”开局之年。我们要全面贯彻省委、省政府和市委的决策部署，突出主题主线，主攻发展重点，强化项目带动，推进区域突破，促进产业提升，加快城镇化进程，坚持民生优先，敢于创新思维，勇于创造优势，善于创建平台，进一步激发动力活力，拓展深化“五大战役”，大干“十二五”开局之年，努力实现科学发展、跨越发展。

　　全市经济社会发展的主要预期目标是：全市生产总值增长15%；财政总收入增长23%，其中地方级财政收入增长26%；全社会固定资产投资增长35%；规模工业增加值增长22%，规模工业总产值增长23.4%；农业总产值增长4%；外贸出口增长20%，实际利用外资增长20%；城镇登记失业率控制在4%以内；社会消费品零售总额增长18%，居民消费价格总水平涨幅控制在4%左右；人口自然增长率控制在7.1‰以内；城镇居民人均可支配收入和农民人均纯收入均增长13%；完成单位生产总值能耗、化学需氧量、氨氮、二氧化硫和氮氧化物年度节能减排任务。

实现上述目标，必须着重做好以下七个方面的工作：

　　（一）打好“五大战役”，大干开局之年

　　打好重点项目建设战役。安排市级以上重点在建项目222个，总投资2609.6亿元，年度投资514亿元。同时实施一批农林水利重大工程。产业方面，加快建设龙海正新橡胶、华安正兴车轮、长泰TOTO卫浴等42个项目；建成腾龙芳烃、鸿一粮油扩建等12个项目；推进光伏及玻璃新材料产业园、奥特莱斯现代服务产业园等88个重点项目前期工作取得突破。交通、能源方面，加快推进沿海大通道建设，开工东山联络线、漳永高速、古武高速、天宝至龙岩蛟洋扩建等四条高速公路，续建古雷港区南9号泊位、龙厦铁路漳州段、厦漳高速扩建工程等18个项目，尽快启动新江东桥建设，动工建设500千伏漳州北变电站，推进漳州军民合用机场、云霄核电等一批项目前期工作。水利、渔港、耕地、造林方面，完成5个重点地区中小河流整治、15座小（一）型水库除险加固、10万人农村饮水安全工程建设任务。加快4个初级水利化县、4条流域水土流失综合治理以及9个县（市、区）山洪灾害防治非工程措施建设步伐。续建新建13个标准化渔港。实施土地整理2.8万亩，完成补充耕地1.6万亩。抓好新一轮造林绿化，全面完成82万亩植树造林任务。

　　打好新增长区域发展战役。集中力量加快古雷港经济开发区、南太武滨海新区、漳州台商投资区、九龙生态经济区四大新增长区域和其他省级以上开发区的开发建设，实施项目322个，总投资超过1458亿元，年度投资304.8亿元以上。14个重点开发区固定资产投资增长不低于35%，工业增加值增长不低于26%，税收收入增长不低于26%。

　　打好城市建设战役。启动“全国文明城市”创建工作，加快提升城市品位。全市实施城建重点项目209个，总投资390.6亿元，年度投资109亿元，其中中心城区实施城建重点项目60个，总投资161.8亿元，年度投资48亿元。加快《漳州市城市总体规划》、《漳州市域城镇体系规划》修编，完成圆山新区规划编制。抓紧启动新行政中心建设前期工作，加快城市中心区建设，进一步提升城市品位。进一步改善旧城，加快延安南、江滨片区改造步伐。新开工建元路、圣王大道、江滨路西延伸段等18个城建道路，新建一批停车场，改善交通拥堵状况。提升市区绿化水平。加快供水、供气、排水设施建设。全面完成污水管网铺设。加快城市架空缆线下地。改善市区内河水循环。加强机动车尾气治理。配置市区高楼消防设施。加大城市管理投入，拓展创卫工作成果。启动数字化城市管理系统建设，提高城市精细化管理水平。动工建设芝山公园，启动九龙江南岸景观带规划建设。加强厦漳基础设施对接，加快推进厦漳快速干道、城际轻轨、海底隧道前期工作，推动厦漳同城化发展。

　　打好小城镇改革发展战役。扎实推进4个省级试点镇建设，同时把9个市级试点镇纳入战役实施范围。加快规划编制，提高规划管理水平。建设一批基础设施、重大产业、房地产业、社会事业、环境整治和其他关系民生的项目，固定资产投资增幅不低于40%。发挥农村土地综合整治、城乡土地增减挂钩、“三旧”改造、城镇户籍制度改革等政策效应，推动产业、人口加速集聚。

　　打好民生工程战役。实施七大类、89个重点民生项目，总投资495亿元，年度投资108.1亿元。教育文化方面，推进中小学校舍安全工程建设，重建454个项目、72万平方米；解决进城务工人员随迁子女就学问题；新建、改扩建57所公办幼儿园；逐步实施中职免费教育政策；健全完善基层公共文化服务体系建设。继续实施“农家书屋”和“乡镇综合文化站”建设工程；筹建市图书馆新馆。医疗卫生方面，为城乡居民提供十项公共卫生免费服务项目；加快建设基层卫生医疗服务体系、市第二医院、市医院外科大楼、漳州市中医院病房大楼改扩建等项目建设；改扩建10家乡镇卫生院和社区卫生服务中心；开展农村孕妇、城市低保孕妇免费产前筛查诊断。强农惠农方面，开展植树造林工程；实施渔业保险、农村住房保险；建设2个县级社会福利中心、10所乡镇敬老院、30所农村幸福园、60个居家养老服务站、1559个自然灾害避灾点；建设村级综合服务场所和农村社区综合维修服务体系；开展农村实用技术远程培训；加大造福工程搬迁、移民后扶和山海协作等扶贫开发力度；实施新一轮农村电网升级改造工程，提高农村电网供电质量，建设1个电气化县、15个电气化镇、55个电气化村。便民生活方面，新建“农家店”180家，配送中心8个，使连锁化超市覆盖所有乡镇；新建或改造集贸市场14个、社区便利店70个；新建年提供上市商品猪万头以上基地8个、年提供上市蛋品十万公斤基地3个，建设200亩以上蔬菜基地8片；投资8.1亿元推进农村家园清洁行动；市区新建30座公厕；建成315亩城市片林；开展26个绿色乡镇的创建工作。和谐平安方面，深入治理“餐桌污染”，建设食品放心工程；深化“平安漳州”创建活动；支持驻漳部队生产生活和开展科技拥军、文化拥军工程。重大社会事业项目方面，积极筹备第十五届省运会，加快推进漳州体育场建设，启动市体育公园建设，筹建游泳跳水馆、重竞技馆、射击馆、网球馆、棒球场、老年人体育活动中心、体育大厦等一批体育设施。各类保障性住房建设和棚户区改造方面，建设各类保障性住房25140套，其中：新增廉租住房1814套；新增发放租赁住房补贴800户；新增公共租赁住房7900套；新建经济适用房736套；新建限价商品住房7700套；城市棚户区改造4000套；垦区危房改造2190套。

　　（二）突出转型升级，加快壮大现代产业

　　增强自主创新能力。全面实施《全民科学素质行动纲要》，积极争创“国家科技进步先进市”和“国家知识产权试点城市”，支持南靖高新区扩区升级，推进节能照明、智能家电2个国家级高新技术产业化基地加快建设。强化国家、省级企业技术中心认定和建设工作，引导光电、食品、钟表、智能家电及智能仪表等产业组建区域技术创新公共服务平台。深入实施“蓝火计划”，加快中国高校技术转移漳州中心、金峰高新技术创业园、漳州软件园、南太武高新技术产业园区、长泰海投科技创业园等一批创业创新基地建设。大力实施标准化战略，深入实施人才强市战略，加快创新人才培育和引进。新增高新技术企业50家，高新技术产业产值达600亿元以上。

　　推进工业扩量提质。制定出台四大主导产业和四个战略性新兴产业具体扶持意见，推进重点产业快速扩张。继续实施工业“千百工程”，投建500万元以上工业项目1200个以上，其中新开工建设项目500个以上，力争年度工业投资突破500亿元，竣工投产企业突破500家，亿元工业企业突破450家。加大品牌创建扶持力度，实施名牌发展战略，鼓励企业争创国家级、省级品牌，推进“一县新创一个中国驰名商标”工作。做好首届漳州市质量奖评选工作，提高企业质量管理水平。健全中小企业融资担保体系建设，帮助中小企业解决融资需求，促进中小企业加快发展壮大。

　　提升现代服务业发展水平。加快建设九大物流园区，积极推进红星美凯龙、吉马汽车城、芗城闽南汽车交易城、平和中国琯溪蜜柚交易中心等一批商贸项目，做大做强现代物流和商贸流通。大力发展金融服务业，引进一批金融机构，支持发展信托、金融租赁、汽车金融、财务公司等非银行金融机构。积极推进智慧城市建设，大力发展信息服务业。扶持中介机构加快发展，支持发展以社区服务、养老服务和病患陪护为重点的家庭服务业。

　　推进旅游业加快发展。加快一批高星级酒店建设进度，力争年内创评2家四星级以上酒店。强化漳州旅游目的地整体形象宣传，发展海峡旅游品牌，加强漳台旅游对接，着力构筑“成片连线、特色鲜明”的漳州旅游发展新格局。积极鼓励各地特色旅游景区创建国家级风景名胜区，巩固提升南靖、华安土楼景区，支持云洞岩风景区、东山生态旅游岛、乌山红色旅游景区、林语堂文化博览园等一批旅游景点加快建设，推进平和灵通山申报国家地质公园。

　　大力发展海洋经济。加强统筹规划，深化岸线资源整合，合理安排产业布局，推进港口建设、口岸建设与临港工业、现代物流、城市建设相衔接，高标准建设一批临港工业、船舶与海洋工程装备、海洋渔业、海洋运输、港口物流和滨海旅游重点项目。推进国家海洋三所海洋科技中试基地建设，支持诏安金都闽南海洋生物医药高科技产业基地发展，加快发展远洋捕捞业，促进海洋生物医药、海洋能开发、海水综合利用、海洋环保等新兴产业发展。科学开发利用无居民海岛。

　　（三）扩大有效需求，激发经济发展动力

　　强化投资引领。强化项目的规划引导、策划生成、储备管理和协调推进，健全项目的台账管理、节点监控和要素保障机制，推进一批产业升级、科技创新、基础设施、民生领域项目加快建设。围绕国家和省项目布局的重点和导向，精心策划生成一批事关长远、事关大局的生产性、基础性、民生性重大项目，推动形成“投产一批、开工一批、储备一批”的滚动发展格局。

　　提升消费需求。落实调整收入分配的政策措施，努力提高居民收入，增强消费能力。继续推进“万村千乡”市场工程，完善城乡流通市场网络。结合新兴城市综合体建设，培育一批餐饮酒店、时尚消费、休闲购物、娱乐健身的商业街区，促进消费结构升级。加强房地产市场调控，引导理性消费，促进房地产市场健康发展。加强服务领域消费维权工作，加强重要商品、居民生活必需品的价格监测和调控保障，优化消费环境。

　　拓展外贸市场。培育壮大国家级光电科技兴贸创新基地和省级农副产品及加工品出口基地、家用电器及钟表出口基地，打造一批具有较强自主创新能力和品牌影响力的大型骨干出口企业。扩大自由贸易区市场，拓展拉美、非洲等新兴市场，推动出口市场多元化。深入开展“品牌兴企”活动，提升出口商品的国际竞争力。用好用活扶持外贸发展的政策，推进出口企业的跨境贸易人民币结算试点工作，实施好有关出口信保、服务贸易等外贸促进政策，支持企业参加各种国际认证。

　　（四）强化基础地位，推进农村发展繁荣

　　大力发展现代农业。稳定粮食播种面积，开展高产创建活动，提高粮食保障能力。加快建设欧中现代农业技术研发中心和海峡两岸农业科技服务中心,大力发展现代种业，加强优质种子种苗引进试验、示范推广。大力发展农产品精深加工，建立10个食品加工示范区，壮大100家食品工业骨干企业，力争产值上10亿元食品龙头企业突破10家。培植一批上规模花卉龙头企业，扩大花卉产业规模。推广生态渔业模式，推进海洋牧场建设。抓好农产品营销，促进农超对接，加快推进海峡两岸农产品物流城、水产品加工集散基地、花卉集散中心及闽荷花卉交流合作中心等重点项目建设。加强农产品地理标志保护和质量安全示范区建设，完善质量安全可追溯制度。实施农民增收“八大工程”，扩宽农民增收渠道。加大对老、少、边、穷地区的扶持力度，加快脱贫致富步伐。

　　加强农村基础建设。加大基本农田保护力度，继续推进土地整理和补充耕地工作，实现耕地占补平衡。建成标准化养殖池塘1万亩、标准渔港10个。加强水利设施建设，开展第一次全国水利普查，推进十大重点水利枢纽工程建设，加快水库除险加固、防洪、海堤、农村饮水安全、中小流域治理等一批水利项目建设。完善重大动植物疫病防控体系，实施农村电网改造升级、路网改造、危房改造、公厕改造、信息化等工程。

　　深化农村改革创新。完善土地承包经营权流转制度，鼓励发展多种形式的适度规模经营。深化集体林权制度改革，扩大林权抵押贷款。推进农业“五新”进村入户，提高农业发展的科技支撑水平。抓好创新农村工作机制，加强农村基层政权建设，完善干部驻村任职、农村“六大员”、科技特派员等制度，探索推进农协会、农民专业合作社建设。做好镇村公益性债务化解工作，健全农业综合信息网络与平台。

　　扎实推进新农村建设。坚持家园规划、家园整理、家园清洁、家园绿化“四位一体”，继续抓好20个市级新农村示范村建设，完成村容整洁120个市级示范村、200个县级重点村建设任务，加快242个村“绿色村庄”创建步伐。推动有条件的地方开展村镇住宅建设试点，促进农村社区化管理。

　　（五）着力先行先试，进一步扩大开放深化改革

　　深化对台交流合作。落实两岸经济合作框架协议，扩大我市优势产品对台出口，增加我市急需的台湾产品进口。深入实施《漳台产业深度对接工作方案》，发挥台湾农工商协会海西联络处作用，促进漳台行业协会建立长效合作机制，力争承接台湾石化、钢铁、装备、光电、电子等产业的整体性转移。全力推进古雷炼化一体化项目前期工作，力争早日获批。继续推进台湾农民创业园扩区升级，拓展漳州台商投资区的品牌与政策效应，争取引进更多台资项目。加强与台湾现代服务业合作，推进我市服务业加快发展。推动台商在漳设立独资医疗机构，开展经纪业务和电子商务合作。更高水平办好第三届农博会暨第十三届花博会。积极承办第三届海峡论坛相关活动，深入开展漳台乡镇对口交流合作，争取招银港开通对台客运直航、东山对台货运直航常态化。深化漳台民间交往交流，加快陈元光漳台文化公园、福建（漳州）闽台文化影视城建设，推动两岸教育合作实验园区建设，推动两岸职业教育、高等教育合作。

　　扩大招商引资成效。着力引进台湾双百企业、央属企业、大型民企、世界500强、华侨华人大财团，力求招商引资实现新突破。坚持以规划引领项目、以产业集聚项目，大力引进高端制造业和战略性新兴产业。深入推进开发区新一轮创业，进一步创新开发区服务机制，推进开发区在跨越发展中发挥排头兵作用。继续深化漳州与香港、澳门的经贸合作，加强与海外华侨华人尤其是华裔新生代、新华侨华人及重点社团的联系。

　　加大改革攻坚力度。大力支持企业上市，推动一批企业进入上市辅导期。积极扩大债券融资、私募股权融资、租赁融资以及BT、BOT等融资，提高直接融资的比重。扩大村镇银行和小额贷款公司试点。鼓励民间资本进入基础设施、公用事业等领域。完成市、县（市、区）两级政府机构改革工作，加快转变政府职能。加快国有企业改制工作，完善国有资产监督管理。加强行政服务中心建设，完善网上审批和电子监察系统，扩大网上并联审批试点，推进公共资源市场化配置，深入规范行政权力运行。

　　（六）加强环境保护，推进生态名市建设

　　强力推进节能减排。严格落实生态环境功能区划，构建生态补偿机制，完善差别化的区域开发配套政策，严把新上项目产业政策、资源消耗和环境保护关。推动节能节水节材节地，实施开发区规划环评，建设生态工业园区。完善激励约束机制，推动节能减排新技术、新装备的研发、引进与应用，推广高耗能产业合同能源管理。严格控制高耗能、高排放项目，坚决淘汰落后产能，着力抓好十大重点领域节能工程项目。加快县城污水处理厂实施脱氮除磷改造和配套管网建设，提高污水收集处理率。大力推行清洁生产，扎实推进循环经济示范试点企业和循环经济示范试点园区建设。

　　深入开展环境整治。着力深化九龙江流域水环境综合治理、重点水库综合整治和农村环境连片整治，强化饮用水源地保护，确保九龙江流域漳州段Ⅲ类水质达标率95%以上，县级以上集中式饮用水源水质达标率100%。扎实推进工业园区环境整治，所有省级以上工业园区都要建成污水集中治理设施。强化日常环境监管，突出抓好重金属污染防治，加强主要河流入海口及重要海湾的海洋环境整治，启动八尺门贯通工程，继续实施“碧海银滩”行动计划，实施沿海五个县（市）海洋生态修复项目工程。完善防范和妥善处置突发环境事件应急机制，保障环境安全。

　　加强生态环境保护。全力抓好新一轮造林绿化工作，着力推进“四绿”工程建设, 启动国家森林城市创建活动，加快建设一批城市森林公园，鼓励支持创建国家级生态县（市）。加强水资源保护开发利用，强化江河、海域采砂监管，加大矿产资源开发整合和保护力度。加强生态功能区保护，强化自然保护区和湿地管理。

　　（七）坚持民生优先，加大保障和改善民生力度

　　扩大就业促进创业。实施积极的就业政策，实现城镇新增就业5.8万人，下岗失业人员再就业1.2万人，农村富余劳动力转移就业6万人。持续改善用工环境，加强就业指导服务，健全就业培训制度。做好大中专毕业生的就业工作，完善城镇就业困难人员就业援助机制，引导农村劳动力合理有序流动。积极推进残疾人居家就业。完善小额贷款和社保补贴政策，鼓励更多劳动者自主创业。健全劳动关系三方协调机制，加大劳动保障监察执法力度，建立和谐劳动关系，保障劳动者实现体面劳动。

　　完善社会保障体系。贯彻落实《社会保险法》，加快完善社会保障体系，扩大社保覆盖面，继续提高社保待遇。探索建立统一的城乡居民养老保险制度，提高企业退休人员养老金水平，完善被征地农民的养老保障制度。积极推进医疗保险关系的衔接与转换工作，实施医疗、工伤生育保险市级统筹。完善城乡低保标准动态调整机制，加大对低收入、弱势群体的帮扶力度，加强法律援助工作。重视发展社会福利、慈善和残疾人事业，成立市慈善总会。落实妇女、儿童发展《纲要》，加快推进市、县（市、区）妇女、儿童活动中心建设，加强妇女、儿童工作，维护妇女、儿童合法权益。

　　推动文化发展繁荣。加强社会主义核心价值体系建设，提高人民群众文明素质和创造活力。出台《漳州文化产业发展规划》，筹建文化创意产业园，培育壮大文化创意产业。广泛开展群众性文化活动，重视文艺精品创作和人才培养。加快闽南文化生态保护实验区建设，加强文物和历史文化名城名镇名街名村保护，积极推进“海上丝绸之路”申遗前期工作，加强重要遗迹、遗址、遗物的收集保护，重视非物质文化遗产的传承与保护，持续推进第三次全国文物普查。加强文化市场管理，推进“扫黄打非”进基层。重视档案、文史等工作。发展哲学社会科学，繁荣新闻出版、广播影视、文学艺术。开展全民健身运动和老年体育活动，提高竞技体育水平。

加快社会事业发展。实施名师培养工程，加强师资队伍建设。落实公办民办并举的办园体制，大力发展学前教育。完善城镇新区教育资源配置，推进义务教育均衡发展，力争198所标准化学校通过验收。支持创建教育强县，加强职教集团和实训基地建设，推进县级职教中心办学规范化建设。支持高校内涵延伸发展，扩大技能型、实用型人才培养规模。推进终身教育，开展社区教育，扶持发展特殊教育。深化医药卫生体制改革，加大疾病控制和卫生监督工作力度，鼓励民资和外资举办医疗机构，加快公立医院医药分开改革步伐，致力解决群众看病贵问题。强化药品安全监管，建立“药械远程电子监管中心”。全面做好人口计生工作，深化综合改革，倡导婚育新风，夯实基层基础，提高出生人口素质，稳定低生育水平，促进人口长期均衡发展。加强统计工作，实施统计四大工程，继续做好第六次全国人口普查工作。加快发展老龄事业，推进养老服务体系建设。

　　维护社会和谐稳定。全面启动“六五”普法及依法治市“三五”规划，提高群众法制意识。加强和创新社会管理，着力解决影响社会和谐稳定的突出问题，确保社会既充满活力又和谐稳定。完善领导干部定期接访群众、机关干部带案下访、矛盾纠纷排查化解等信访工作制度，加快维稳“三支队伍”建设，引导群众合理表达诉求，妥善处理和化解社会矛盾。加强未成年人思想道德教育。推进社会管理创新，推动社区矫正工作制度化。健全社会治安防控体系，推动新一轮“平安漳州”建设向更高层次迈进。全力推动企业主体责任的落实，深入开展专项整治和隐患排查，促进安全生产形势持续稳定。健全突发公共事件预警和应急处置机制。加强综合应急救援队伍建设，强化国防教育、国防动员、民兵预备役、海防、人防工作，深化“双拥”共建活动，落实拥军优属政策，实现全国双拥模范城“五连冠”。加强民族宗教工作，依法管理宗教事务。

　　四、加强政府自身建设，提高推动跨越发展的能力

　　各级政府要牢记责任重于泰山、使命高于一切，自觉适应形势的发展变化，进一步加强政府自身建设，着力增强学习力、创新力、执行力、公信力，做到履职有能、创新有方、工作有为、施政有效，在推动跨越发展中争取更大作为。

　　勤学勤思促跨越。增强“知识恐慌”、“本领恐慌”的意识，大力推进学习型政府建设，完善学习制度，拓展学习领域，增强学习实效，促进政府工作人员加快知识更新、优化知识结构，切实提高推动跨越发展的能力与水平。坚持学习与思考并重，应用所学知识深入研究发展中存在的重点难点问题，把学习成果转化为推动跨越发展的思路办法。

　　创新创造促跨越。坚持在干的过程中解放思想，以创新的思维想问题，用改革的办法破难题，靠市场的机制激活力。大力弘扬敢于负责、敢于突破的作风，该负的责任勇于担当，该管的事情主动管好，该解决的问题及时解决，该突破的工作大胆突破。勇于自我超越、追求卓越，不满足于完成常规工作任务，对重点工作升格以求、争创一流，不断释放推动跨越发展的潜能。

求实求效促跨越。树立强化执行、提高效能的理念，对部署的工作快速落实，对认准的事情扭住不放，对重要的事项始终盯紧，形成重执行、求实效的鲜明导向。严格实行层级负责制，健全执行体系，优化工作流程，确保政令畅通，防止执行力层层递减。切实增强运用和落实政策的能力，找准政策与本地本部门工作的结合点，充分用好用足用活各种政策，不断扩大政策效应。真诚关心爱护基层干部，努力为基层排忧解难，保护好基层工作积极性。

　　依法依规促跨越。依法接受人大及其常委会的法律监督、工作监督和政协的民主监督，主动接受社会舆论和人民群众的监督，确保行政权力正确行使。完善行政执法责任制和行政复议、行政赔偿、行政补偿制度，加快推进法治政府建设。健全公众参与、专家论证和政府决策相结合的决策机制，提高决策的科学化、民主化水平。自觉运用法律手段管理经济和社会各项事务，切实维护群众的合法权益。

　　勤政廉政促跨越。坚持从严治政，加大治庸治懒力度，促进勤廉兼优。严格行政纪律，落实岗位职责，完善绩效管理，强化行政问责，惩处渎职失职，促进政府工作人员履职到位、尽责到位。全面加强效能督查，严格落实责任追究，切实解决一些工作人员不在岗、不在状态以及不作为、乱作为的问题。严格落实党风廉政建设责任制，着力加强制度建设，完善惩治和预防腐败体系，加大民主评议行风力度，促进政风行风进一步好转。

各位代表，五年看头年，开局最关键。让我们紧密团结在以胡锦涛为总书记的党中央周围，以邓小平理论和“三个代表”重要思想为指导，深入贯彻落实科学发展观，在省委、省政府和市委的正确领导下，紧紧依靠全市人民，振奋精神、凝心聚力，奋发有为、扎实工作，加快推进漳州科学发展、跨越发展，以优异的成绩迎接中国共产党成立90周年！
